# Complete Response of Para-Aortic and Lateral Pelvic Lymph Node Recurrence of Rectal Cancer Treated to S-1 Monotherapy

**DOI:** 10.4021/wjon619w

**Published:** 2013-03-06

**Authors:** Tomonori Miyazawa, Norihiko Koide, Nobuhiro Fujita

**Affiliations:** a Department of Surgery, Joetsu General Hospital, Joetsu, Japan

**Keywords:** Para-aortic and lateral lymph node recurrence, Rectal cancer, S-1

## Abstract

This report presents a case of para-aortic and lateral pelvic lymph node recurrence of rectal cancer that showed complete response to S-1 monotherapy. A 69-year-old man underwent low anterior resection for rectal cancer in 2007. Para-aortic lymph and right lateral pelvic lymph node recurrence occurred in 2008. He received a fluorouracil/folinic acid plus oxaliplatin regimen; however, G4 neutropenia and G3 fatigue were experienced. We started S-1 monotherapy as a salvage treatment. Abdominal computed tomography did not reveal any para-aortic and lateral pelvic lymph nodes recurrence after 10 cycles of S-1 monotherapy. Hence, response in this case was classified as a complete response. No recurrence was noted 36 months after the complete response. S-1 monotherapy is likely to be effective in treating patients with metastatic colorectal cancer who do not respond to standard combination chemotherapy.

## Introduction

The introduction of oxaliplatin and irinotecan into combination regimens with fluorouracil (5-FU) has been a major advance in the treatment of patients with metastatic colorectal cancer (MCRC). Oxaliplatin/5-FU/folinic acid (FOLFOX-4) is the standard first-line chemotherapy in patients with MCRC [1, 2]. The common grade 3/4 adverse events associated with FOLFOX-4 are neutropenia and granulocytopenia; therefore, treatment might be discontinued because of such adverse events in patients treated with FOLFOX-4 [1, 2]. S-1 is an oral anticancer agent containing tegafur, a metabolically activated prodrug of 5-FU, and 2 biochemical modulators [3]. Three phase 2 trials reported that S-1 monotherapy showed response similar to that of infusional 5-FU/folinic acid as the first-line treatment in patients with MCRC [4-6]. Two studies showed that salvage S-1 monotherapy was effective and well tolerated in MCRC patients after the failure of irinotecan-based or oxaliplatin-based chemotherapy [7, 8]. Here, we report a case of para-aortic and lateral pelvic lymph node recurrence of rectal cancer that showed complete response (CR) to S-1 monotherapy after discontinuance of FOLFOX-4 because of grade 4 adverse events.

## Case Report

**Figure 1:**
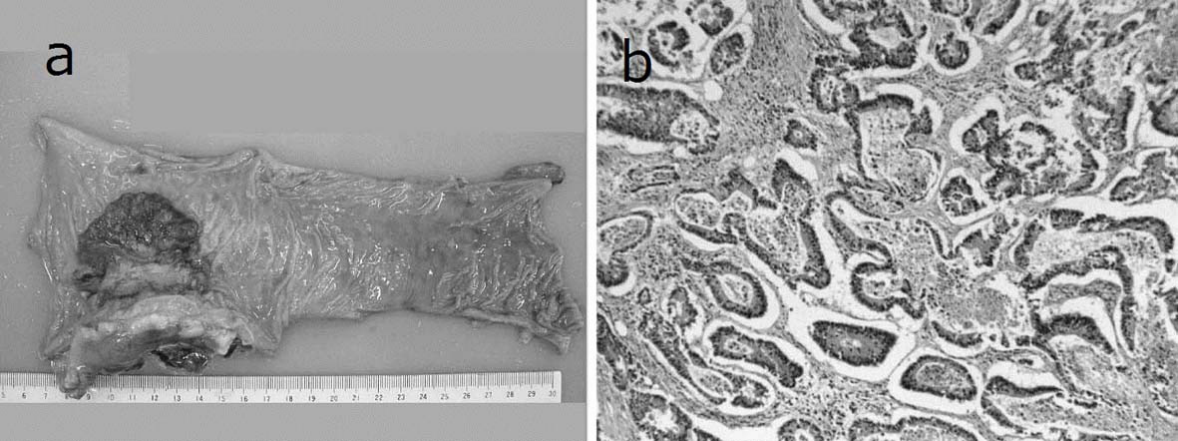
Resected specimen and pathological findings. a): Macroscopic appearance of surgically resected specimen showing type 2 advanced rectal cancer; b): Pathological examination showed a well-differentiated adenocarcinoma invading the perirectal tissues, with metastatic involvement in 1 of the 15 resected lymph nodes. (hematoxylin and eosin stain).

**Figure 2:**
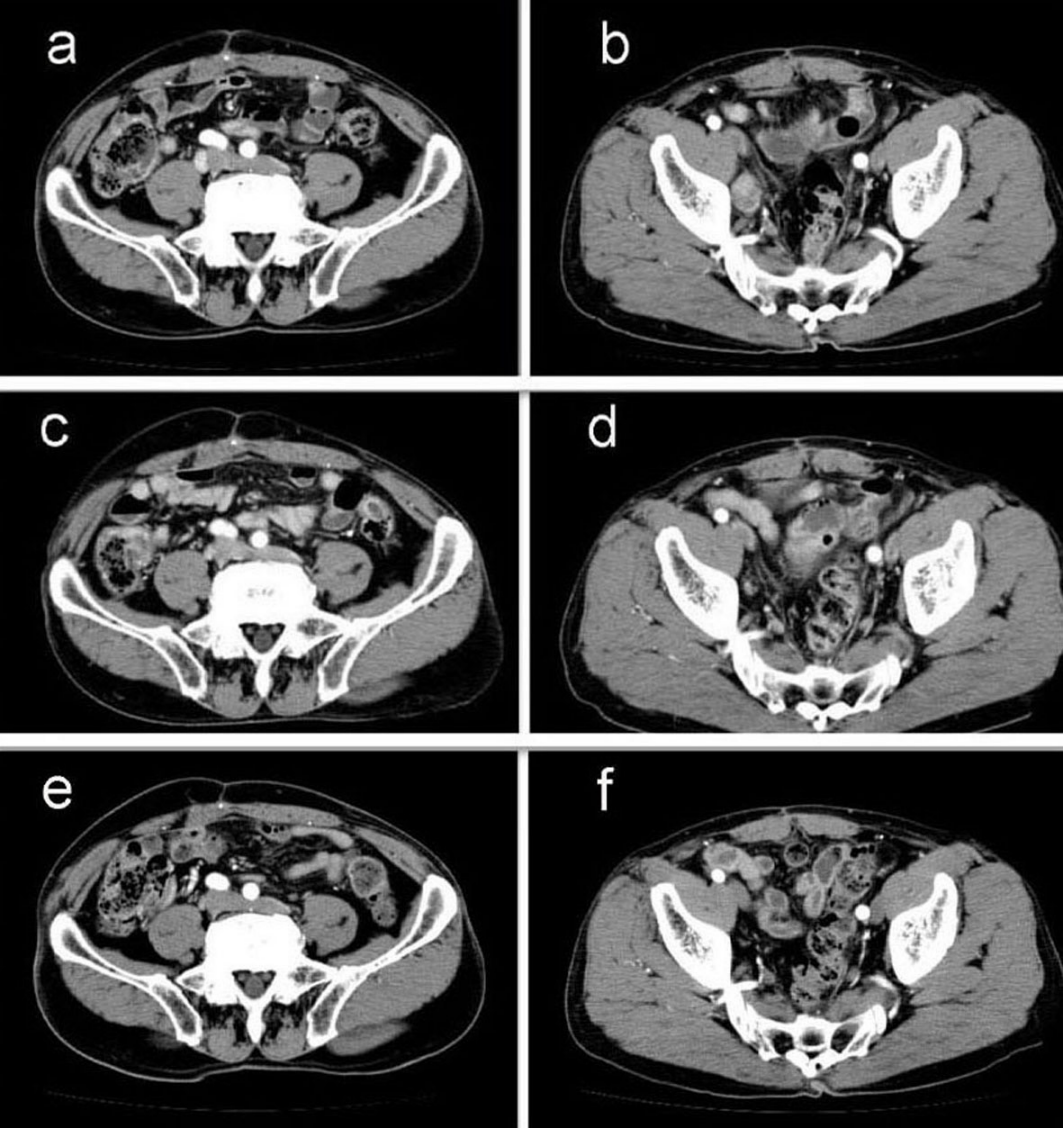
Abdominal computed tomography (CT) findings. a-b): CT scan before chemotherapy showing swollen para-aortic and right lateral pelvic lymph nodes; c-d): CT scan after 5 cycles of S-1 administration showing reduction in the swollen para-aortic lymph nodes; e-f): CT scan after radiation and 10 cycles of S-1 administration showing complete disappearance of swollen para-aortic and lateral pelvic lymph nodes. Hence, response in this case was classified as a complete response.

A 69-year-old man with mild liver dysfunction due to chronic type C hepatitis, underwent low anterior resection for rectal cancer in 2007. Pathological examination showed a well-differentiated adenocarcinoma invading the perirectal tissues, with metastatic involvement in 1 of the 15 lymph nodes removed (T4 N1M0/Stage IIIB), (Fig. 1). The patient underwent 5 cycles of tegafur-uracil plus oral leucovolon therapy (UFT/LV) as adjuvant chemotherapy without any adverse events. In 2008, abdominal computed tomography (CT) after UFT/LV showed swollen para-aortic and right lateral pelvic lymph nodes (Fig. 2a, b). Thus, para-aortic and pelvic lymph nodes recurrence of rectal cancer was diagnosed. We started a FOLFOX-4 regimen in March 2008. However, the patient experienced G4 neutropenia and G3 fatigue (according to the Common Terminology Criteria for Adverse Events Version 4.0), and therefore had to discontinue FOLFOX-4. We started S-1 administration (100 mg/day twice on days 1 - 14, every 3 weeks) as salvage treatment in May 2008. Abdominopelvic CT after 5 cycles of S-1 showed reduction of the swollen para-aortic lymph nodes (Fig. 2c, d).

Another series of abdominal CT performed after 10 cycles of S-1 monotherapy showed complete disappearance of swollen para-aortic and lateral pelvic lymph nodes (Fig. 2e, f). Hence, response in this case was classified as a CR. The patient did not experience any adverse events during the S-1 monotherapy. No signs of recurrence or metastasis were noted 36 months after CR was confirmed.

## Discussion

The key drug for systemic treatment of colorectal cancer is 5-FU. The introduction of oxaliplatin and irinotecan into combination regimens with 5-FU has been a major advance in treatment of patients with MCRC. FOLFOX-4 and irinotecan/5-FU/folinic acid (FOLFIRI) regimens are the standard first-line or second-line chemotherapies in patients with MCRC. However, these regimens involve continuous infusions of 5-FU that requires catheter replacement and regular visits to the clinic [2]. Grade 3/4 neutropenia is more common with FOLFOX-4 than with 5-FU/ folinic acid [1, 2]. Oral 5-FU prodrugs capecitabine and UFT/LV have already been proven to show an efficacy equivalent to that of an intravenous 5-FU/folinic acid regimen in patients of MCRC, but with less toxicity than the 5-FU regimen [9-11]. Furthermore, capecitabine plus oxaliplatin therapy was non-inferior to FOLFOX-4 as a first-line treatment for MCRC [2]. These oral 5-FU prodrugs have the advantage of reducing intravenous drug administration and associated visits to the clinic.

S-1 is an oral anticancer agent containing tegafur, a metabolically activated prodrug of 5-FU, with 2 biochemical modulators of 5-FU metabolism: 5-chloro-2, 4-dihydroxypyridine that inhibits 5-FU degeneration by dihydroxypyridine dehydrogenase and potassium oxonate that reduces the incidence of 5-FU-induced gastrointestinal side effects [3]. Three phase2 trials have demonstrated that S-1 achieved responses similar to those of infusional 5-FU/folinic acid and had acceptable toxicity profile as first-line treatment in patients with MCRC [4-6]. Shibahara et al reported equivalent efficacy and safety between S-1 and UFT/LV in patients with MCRC [12]. Two studies showed that salvage S-1 monotherapy was effective and well tolerated in MCRC patients after failure of irinotecan-based or oxaliplatin-based chemotherapy [7, 8]. Recent studies demonstrated that the combination of S-1 and oxaliplatin or irinotecan could be an additional therapeutic options for patients with MCRC [13-15]. S-1 is usually administrated for 4 weeks, followed by a 2-week drug-free period. However, adverse reactions related to S-1 therapy commonly begin to appear in 2 - 3 weeks after treatment starts. Therefore, a 2-week regimen of S-1 followed by a 1-week drug- free period might mitigate adverse reactions and prolong the medication period [16]. Our patient did not experience any severe adverse events during 10 cycles of the 2-week regimen of S-1.

In conclusion, we experienced a case of para-aortic and lateral pelvic lymph node recurrence of rectal cancer that showed CR to salvage S-1 monotherapy after the failure of a FOLFOX-4 regimen. S-1 is likely to be an effective, well-tolerated and convenient therapeutic option for patients with advanced colorectal cancer.
